# Species-specific responses during Seoul orthohantavirus infection in human and rat lung microvascular endothelial cells

**DOI:** 10.1371/journal.pntd.0012074

**Published:** 2024-03-27

**Authors:** Danny Noack, Mirjam C. G. N. van den Hout, Carmen W. E. Embregts, Wilfred F. J. van IJcken, Marion P. G. Koopmans, Barry Rockx

**Affiliations:** 1 Department of Viroscience, Erasmus University Medical Center, Rotterdam, the Netherlands; 2 Department of Cell Biology, Erasmus University Medical Center, Rotterdam, the Netherlands; 3 Center for Biomics, Erasmus University Medical Center, Rotterdam, the Netherlands; Karolinska Institutet, SWEDEN

## Abstract

Seoul orthohantavirus (SEOV) is a rat-borne zoonotic virus that is transmitted via inhalation of aerosolized infectious excreta, and can cause hemorrhagic fever with renal syndrome (HFRS) in humans worldwide. In rats, SEOV predominantly exists as a persistent infection in the absence of overt clinical signs. Lack of disease in rats is attributed to downregulation of pro-inflammatory and upregulation of regulatory host responses. As lung microvascular endothelial cells (LMECs) represent a primary target of infection in both human and rats, infections in these cells provide a unique opportunity to study the central role of LMECs in the dichotomy between pathogenicity in both species. In this study, host responses to SEOV infection in primary human and rat LMECs were directly compared on a transcriptional level. As infection of rat LMECs was more efficient than human LMECs, the majority of anti-viral defense responses were observed earlier in rat LMECs. Most prominently, SEOV-induced processes in both species included responses to cytokine stimulus, negative regulation of innate immune responses, responses to type I and II interferons, regulation of pattern recognition receptor signaling and MHC-I signaling. However, over time, in the rat LMECs, responses shifted from an anti-viral state towards a more immunotolerant state displayed by a PD-L1, B2M-, JAK2-focused interaction network aiding in negative regulation of cytotoxic CD8-positive T cell activation. This suggests a novel mechanism by which species-specific orthohantavirus-induced endothelium and T cell crosstalk may play a crucial role in the development of acute disease in humans and persistence in rodents.

## Author summary

Seoul orthohantavirus is a rat-borne zoonotic virus that can cause hemorrhagic fever with renal syndrome in humans worldwide while it generally leads to persistent infection in absence of clinical signs in the natural reservoir host, the Norway rat. It is believed that persistent infection in reservoir hosts is due to downregulation of pro-inflammatory immune responses and upregulation of regulatory responses, while in humans excessive immune responses contribute to disease. Endothelial cells in the lungs are of particular interest as they represent a primary target during Seoul orthohantavirus infection in both species. So far, it remains incompletely understood how the responses of these cells to Seoul orthohantavirus contribute to the development of disease in humans and the prevention of such in rats. In this study, we compared the host responses of human and rat lung endothelial cells to Seoul orthohantavirus infection and found that anti-viral responses from both species are comparable, but differ in timing. Additionally, we identified a novel protein interaction network during later phases of acute infection in rat cells, which may play a crucial role in the development of disease in humans and persistence in rodents.

## Introduction

Orthohantaviruses are predominantly rodent-borne zoonoses able to cause disease of varying severity in humans, depending on host susceptibility and causative virus species. These viruses are wide-spread with each virus species existing in a specific rodent reservoir species in which they generally do not cause overt disease. Seoul orthohantavirus (SEOV) is considered the most widely distributed orthohantavirus as SEOV is predominantly detected in the omnipresent Norway rat (*Rattus norvegicus)*. Upon inhalation of aerosolized excreta, humans can become infected and develop hemorrhagic fever with renal syndrome (HFRS). This syndrome is characterized by flu-like symptoms such as fever, myalgia, malaise and more specific symptoms including acute kidney injury, proteinuria, hematuria and thrombocytopenia [1]. The number of reports describing SEOV infections in pet rats, rat owners and rat breeding farms is increasing worldwide, demonstrating the relevance of its zoonotic potential [2–6]. It is hypothesized that upon inhalation of virus particles, the lungs are a primary site of virus replication in both human and rat [7,8]. A central feature of orthohantavirus infection in humans and rats is nonlytic infection of endothelial cells of different organs, including lungs and kidneys [4,7–11]. Previous studies have suggested that persistent infection in reservoir hosts is due to selective downregulation of pro-inflammatory immune responses and upregulation of regulatory responses allowing ongoing viral replication in the absence of immunopathology [7,12–15]. The human immune response upon orthohantavirus infection is excessive compared to that of the reservoir host species and is hypothesized to be directly linked to the pathogenicity [16]. Enhancement of programmed death-ligand 1 (PD-L1) expression by rat endothelial cells, with subsequent inhibition of CD8-positive (CD8^+^) and induction of regulatory T cells during SEOV infection is hypothesized to explain this drastic difference in pathogenicity [15]. However, an in depth comparison between host responses of human and rat endothelial cells to acute SEOV infection, with a potential central role for PD-L1 and associated factors, remains to be executed. Therefore, this study aims to identify the molecular mechanisms of SEOV pathogenesis by comparing host responses to SEOV infection in primary human and rat lung microvascular endothelial cells (LMECs) through transcriptome analyses. By comparing host responses in humans to those in rodents, this study provides insight into mechanisms that lead to disease in humans and prevent pathological outcomes in rodents.

## Methods

### Cell culture

Commercially available primary cultures of Sprague-Dawley rat, inbred laboratory strain of *Rattus norvegicus*, lung microvascular endothelial cells (LMECs, Cell Biologics) and primary human LMECs (Cell Systems) were propagated in endothelial cell growth medium MV2 (PromoCell) supplemented with 100 IU/ml penicillin and 100 μg/ml streptomycin (Lonza). Primary LMECs were grown on 1% gelatin (Sigma-Aldrich) pre-coated tissue culture flasks and plates at 37°C in a humidified CO_2_ incubator. Experiments were performed with primary cells up to passage 10. Vero E6 cells (CRL-1586, ATCC) were cultured in Dulbecco’s modified Eagle’s medium (DMEM, Capricorn Scientific) supplemented with 10% fetal calf serum (FCS), HEPES, sodium bicarbonate, 100 IU/ml penicillin and 100 μg/ml streptomycin (Lonza) at 37°C in a humidified CO_2_ incubator. All cells and virus stocks were confirmed to be free of mycoplasma.

### Virus infections

Seoul 80–39 orthohantavirus (SEOV, European Virus Archive Global #008v-EVA1473, P+2) stocks were propagated on Vero E6 cells in DMEM with 5% FCS, HEPES, sodium bicarbonate, 100 IU/ml pen/strep at 37°C in a humidified CO_2_ incubator. Culture supernatants were collected 9 days post-infection (dpi), concentrated with 100 kDa Amicon Ultra centrifugal filter units (Merck Millipore) and aliquots were stored at -80°C. Virus was quantified by 50% tissue culture infectious dose (TCID_50_) dilution assays. In brief, ten-fold dilutions of infectious supernatants were added in triplicate to 96-wells plates (Greiner) with Vero E6 cells and incubated for five days at 37°C in a humidified CO_2_ incubator. Subsequently, cells were fixed with ice-cold absolute ethanol and 70% ethanol. After washing thrice with phosphate-buffered saline (PBS), cells were blocked with 5% dried milk powder (Campina) in PBS. Detection of SEOV nucleoprotein (N)-positive cells was performed using a cross-reactive mouse monoclonal antibody TULV 1 (1:50) [17] and a donkey anti-mouse Alexa Fluor 488-conjugated secondary antibody (1:500, Invitrogen) using an Axio Observer Vert. 1A fluorescent microscope (Zeiss). TCID_50_/ml values were calculated according to methods described by Spearman & Kärber. To determine virus replication kinetics, the same number of LMECs for both species were inoculated for 2 hours with SEOV at a multiplicity of infection (MOI) of 0.1 or 1.0. For other experiments, unless stated otherwise, a MOI of 1.0 was used. Two hours post-infection, cells were washed thrice with PBS, and provided with fresh endothelial cell culture medium, followed by medium refreshing every two days. All infection experiments were performed in a class II Biosafety Cabinet under biosafety level 3 (BSL-3) conditions at Erasmus MC.

### Immunofluorescence staining

LMECs were fixed, blocked and stained according to the abovementioned protocol as used for Vero E6 cells. Additionally, after final three washing steps with PBS, Hoechst 33342 (ThermoFisher) solution was added before samples were washed one last time and kept in PBS and stored at 4°C until imaging. Immunofluorescent samples were imaged at 40x magnification using an Axio Observer Vert. 1A fluorescent microscope with ZEN software (Zeiss). Four representative images per well were taken. For image visualizations ImageJ software was used. Train object classifier on QuPath software with manual confirmations were utilized to quantify infection percentages of LMECs of both species.

### RNA isolation and RT-qPCR

For reverse transcription quantitative PCR (RT-qPCR) and RNA-Sequencing (RNA-Seq), primary human and rat LMECs were either inoculated in triplicate with SEOV at a MOI of 1.0 or mock-infected, which consisted of either SEOV stock or equal volume of virus propagation medium diluted in endothelial cell culture medium. For RT-qPCR, additional triplicates were stimulated with 10 μg/ml polyinosinic:polycytidylic acid (Poly I:C, Invivogen) 24 hours prior to RNA collection at 4 and 12 dpi serving as positive controls. Total RNA samples from primary LMECs were collected and isolated via the high pure RNA isolation kit (Roche) according to manufacturer’s guidelines. RT-qPCR was performed on a 7500 Real-Time PCR System (Applied Biosystems). Commercially available Taqman primer-probe mixes were used for gene expression of human MX-dynamin-like GTPase 1 (*MX1*; Hs008956008_m1), rat *Mx1* (Rn00597360_m1), human programmed death-ligand 1 (*CD274*; Hs00204257_m1), rat *Cd274* (Rn01760313_m1) and rat beta-actin (*Actb*; Rn00667869_m1). Human *ACTB* was amplified using in-house designed primer-probe sets: *ACTB*_Fw GGCATCCACGAAACTACCTT, *ACTB*_Rv AGCACTGTGTTGGCGTACAG, *ACTB*_Probe FAM-ACCACAGTCCATGCCATCACTGCCA-BHQ1. Host gene expressions were normalized to beta-actin gene of either rat or human origin. Relative host gene expressions were calculated via the 2^-ΔΔCT^ method [18].

### RNA-Seq library prep

Total RNA was checked for quality on an Agilent Technologies 2100 Bioanalyzer using a RNA nano assay. All samples had RIN values greater than 7.5. RNA-Seq libraries were prepared according to the Illumina TruSeq stranded mRNA protocol. Briefly, 200 ng of total RNA was purified using poly-T oligo-attached magnetic beads to end up with poly-A containing mRNA. The poly-A tailed mRNA was fragmented and cDNA was synthesized using SuperScript II and random primers in the presence of Actinomycin D. cDNA fragments were end repaired, purified with AMPure XP beads (Beckman Coulter) and A-tailed using Klenow exo-enzyme in the presence of dATP. Paired end adapters with dual index (Illumina) were ligated to the A-tailed cDNA fragments and purified using AMPure XP beads. The resulting adapter-modified cDNA fragments were enriched by PCR using Phusion polymerase as followed: 30 s at 98°C, 15 cycles of (10 s at 98°C, 30 s at 60°C, 30 s at 72°C), 5 min at 72°C. PCR products were purified using AMPure XP beads and eluted in 30 μl of resuspension buffer. One microliter was loaded on an Agilent Technologies 2100 Bioanalyzer using a DNA 1000 assay to determine the library concentration and for quality check. The libraries were sequenced for single-end reads, 50bp in length, on Illumina HiSeq2500 in Rapid run mode. At least 20M reads were generated for each library.

### RNA-Seq primary analysis

Cutadapt [19] was used to trim off Illumina adapter sequences from the reads, which were subsequently mapped against the reference using HiSat2 version 2.2.1 [20]. For the human libraries, GRCh38 reference genome was used, and for the rat libraries, mRatBN7-2. Gene expression values were called using htseq-count version 0.12.4 [21], Ensembl release 101 (human) and 105 (rat) gene and transcript annotation. When rat gene names remained unavailable, corresponding gene names, in case available, were manually assigned by accessing the Rat Genome Database [22].

### Differential gene expression

Sample QC and differential expression analysis were performed in the R environment for statistical computing version 3.6.3 [23], using the packages DESeq2 version 1.26.0 [24] and tidyverse version 1.3.0 [25]. Throughout the study, significantly upregulated gene expressions were defined as -Log_10_P ≥ 2 with Log_2_ fold change > 1 while significantly downregulated gene expressions were defined as -Log_10_P ≥ 2 with Log_2_ fold change < -1. The top upregulated gene expressions were defined as the highest fold change of significantly altered (-Log_10_P ≥ 2) gene expressions and the top downregulated gene expressions were defined as the lowest fold change of significanlty altered (-Log_10_P ≥ 2) gene expressions. The Venn diagram for significantly differentially expressed genes was created using InteractiVenn [26].

### Pathway enrichment analysis

The web-based gene annotation and analysis tool Metascape version 3.5 was utilized for pathway enrichment analysis (accessed on January 24^th^ 2023) [27]. Input species *Homo sapiens* and *Rattus norvegicus* were selected and GO-terms for molecular functions, biological processes and cellular components were utilized with a minimum overlap of 3, a P-value cut-off of 0.01 and a minimum enrichment of 1.5. For comparison of enriched GO-terms as response to SEOV infection in both species, cut-offs LogP > 5 and GeneRatio > 0.05 were utilized. Web-based gene ontology database AmiGO 2 version 2.5.17 was utilized to identify species-specific protein-encoding genes included for each relevant GO-term [28]. STRING database version 11.5 was utilized for generation of functional protein association networks with minimum required interaction score of 0.400 [29].

### Data visualization

GraphPad Prism 9 was used for heatmaps, R with ggplot and EnhancedVolcano for data visualizations. Adobe Illustrator was used to combine data visualizations into final figures. Biorender was utilized for creating images of human and rat.

### Statistical analysis

Statistical analyses were performed using GraphPad Prism 9 software. Viral titers were log-transformed and subsequently compared by two-way ANOVA followed by Sidak-multiple comparison test. Relative gene expression levels of log-transformed human *MX1* and rat *Mx1*, and human *CD274* and rat *Cd274* were compared to each mock condition on the particular time point and statistical significance was determined by two-way ANOVA with Dunnett’s multiple comparison test. All results are expressed as means with a standard error of the mean (SEM) with P < 0.05 considered statistically significant.

## Results

### More efficient SEOV replication in LMECs of rat origin compared to human

**Fig 1:**
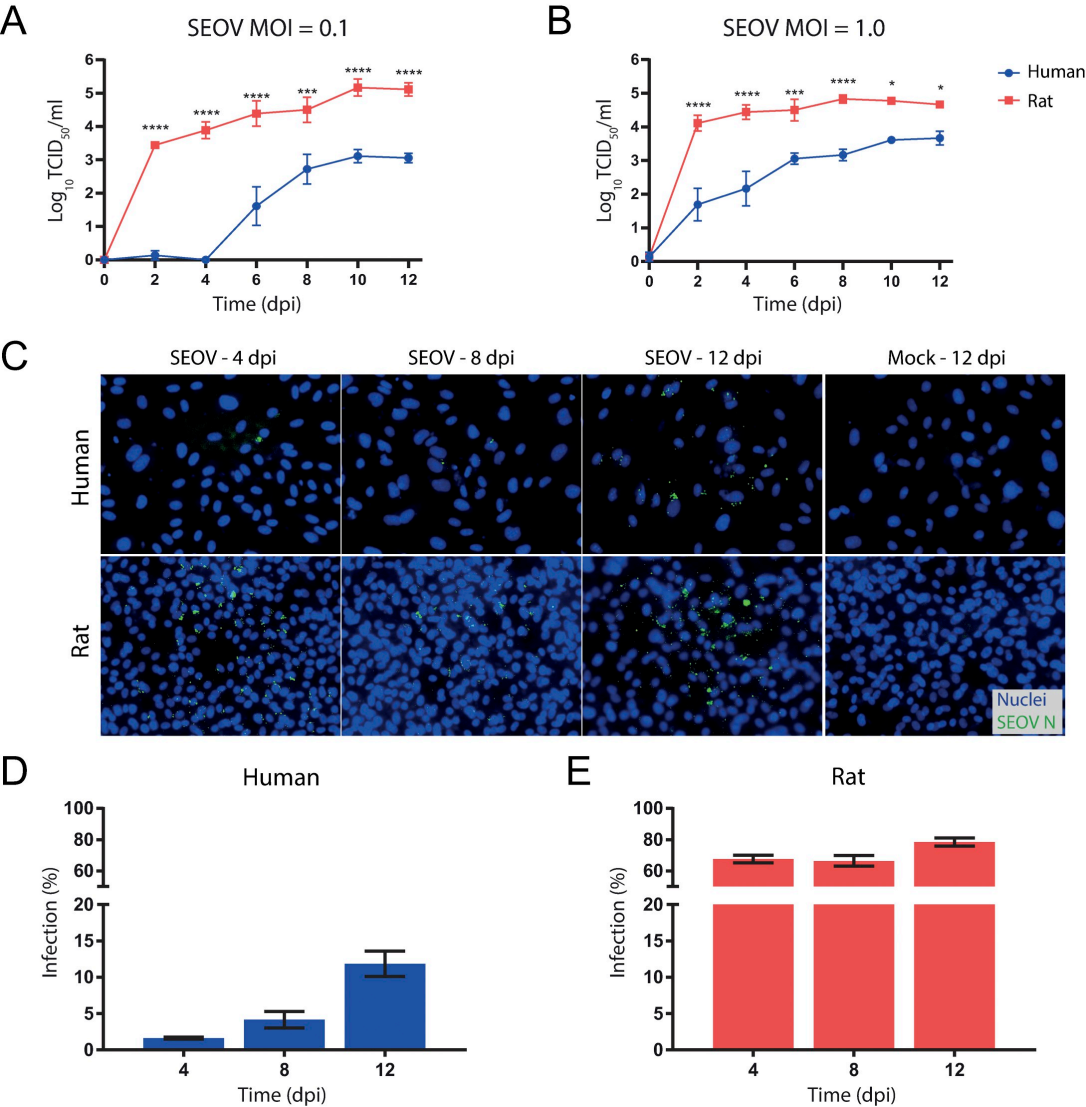
SEOV infection of human and rat lung microvascular endothelial cells (LMECs). (**A** and **B**) Live virus titers can be detected by virus titrations on Vero E6 cells of supernatant taken at every two days. Error bars indicate SEM with results from two independent experiments performed with each three biological replicates per condition. Statistical significance was determined by two-way ANOVA followed by Sidak-multiple comparison test. *P < 0.05, ***P < 0.001, ****P < 0.0001. (**C**) Immunofluorescence staining of SEOV-infected human and rat LMECs (MOI of 1.0) at 4, 8 and 12 days post-infection (dpi) by staining for viral nucleoprotein (green) and nuclei (blue). Images at 40x magnification are representative of two independent experiments with each three biological replicates per condition. Infected human (**D**) and rat (**E**) LMECs were quantified by immunofluorescent signal for SEOV N. Error bars indicate SEM with results from two independent experiments.

To determine whether SEOV replicates differently in human versus reservoir target cells, we cultured human and rat LMECs under identical conditions. Several important characteristic differences between human and rat LMECs include that rat LMECs are much smaller in size and display less contact-inhibition when cultured for longer periods of time. Initial observations on the proliferation of these cells, suggested that rat LMECs proliferate faster (possibly due to the higher metabolic rate), compared to human LMECs. Both cell types grew to confluency within two days post-seeding. Then, LMECs of human and rat origin were infected with SEOV at a low MOI (0.1, Fig 1A) or high MOI (1.0, Fig 1B). Upon infection with a low MOI, SEOV infectious titers were first detected in supernatant of human LMECs by 6 dpi, reaching a peak titer of 1.29*10^3^ TCID_50_/ml by 10 dpi. In rat LMECs viral titers were detected earlier (2 dpi) and SEOV replicated to significantly higher titers compared to human LMECs, throughout the duration of the experiment with peak titers of 1.47*10^5^ TCID_50_/ml by 10 dpi (Fig 1A). Similar kinetics were observed upon infection with high MOI, although earlier detection of SEOV titers by 2 dpi was observed in human LMECs compared to low MOI, peaking by 12 dpi, with titers of 4.64*10^3^ TCID_50_/ml. SEOV titers in rat LMECs infected with a high MOI increased as early as 2 dpi, and remained high throughout the experiment with peak titers of 6.81*10^4^ TCID_50_/ml (Fig 1B). To determine the number of infected cells in LMECs infected with a high MOI, SEOV nucleoprotein (N)-positive cells were detected (Fig 1C) and infection percentages increased over time for human LMECs from approximately 2% on 4 dpi to 12% on 12 dpi. The number of SEOV N-positive cells was substantially higher in rat LMECs, increasing from 68% on 4 dpi to 78% on 12 dpi (Fig 1D). These data demonstrate that LMECs of both human and rat origin were susceptible for infection with SEOV. However, SEOV infected and replicated more efficiently and to higher titers in rat LMECs compared to human LMECs.

### Differential regulation of gene expressions in SEOV-infected LMECs from human and rat

**Fig 2:**
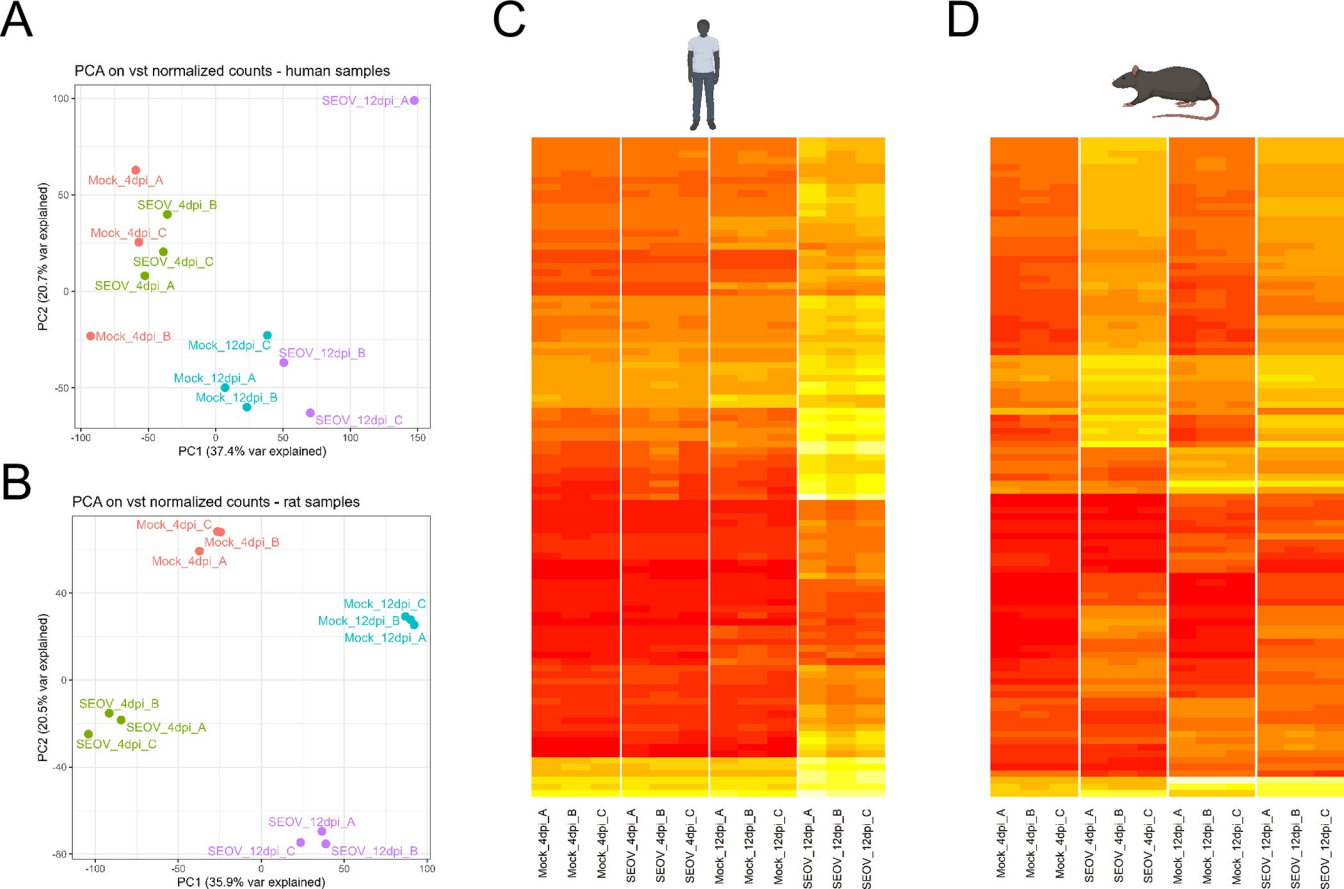
RNA-Seq analyses of SEOV-infected and mock-infected human and rat LMECs at 4 and 12 days post-infection (dpi). (**A**) Principal component analysis (PCA) plot based on vst normalized counts of human samples demonstrating 37.4% of the variance explained by PC1 and 20.7% by PC2. (**B**) PCA plot based on vst normalized counts of rat samples demonstrating 35.9% of the variance explained by PC1 and 20.5% by PC2. (**C**) Heatmaps of the top 100 most variable genes in human samples on 4 and 12 dpi. (**D**) Heatmaps of the top 100 most differentially variable genes in rat samples on 4 and 12 dpi. Biorender was used for creating images of human and rat.

To compare host gene response kinetics of human and rat LMECs during SEOV infection, two different time points were selected for bulk RNA-sequencing (RNA-Seq) analyses. The first time point was selected as an early time point where viral titers in supernatants of both species were detectable and increasing (4dpi, Fig 1B). The second time point was selected as a later time point where replication kinetics had plateaued for both species (12 dpi, Fig 1B). Principal component analysis (PCA) illustrated clustering of biological replicates within the different cell types and treatment groups and demonstrated a profound transcriptomic shift in response to infection (Fig 2A and 2B). For human LMEC samples, no separate clustering between mock and SEOV-infected human samples was observed at 4 dpi (Fig 2A). This indicated that the variation in the bulk RNA-Seq profiles between mock and SEOV infection at 4 dpi is of the same order of magnitude as the variation between replicates within these groups. This was also illustrated by a heat map of the top 100 most variable genes (Fig 2C), and even more by the complete lack of any significantly altered genes between these conditions at 4 dpi (S1 Fig). Therefore, time point 4 dpi for human LMEC samples was not taken along in further downstream analyses within this study. The PCA plot for rat samples provided clear separation of mock and SEOV-infected samples at both time points (Fig 2B). Clear clusters of the top 100 most differentially expressed genes were detected in SEOV-infected rat LMECs for both time points (Fig 2D). These findings indicate that SEOV infection induced distinct host gene expression profiles in human LMECs at 12 dpi, but not at 4 dpi, and in rat LMECs at both time points.

### Anti-viral responses in human LMECs upon SEOV infection

**Fig 3:**
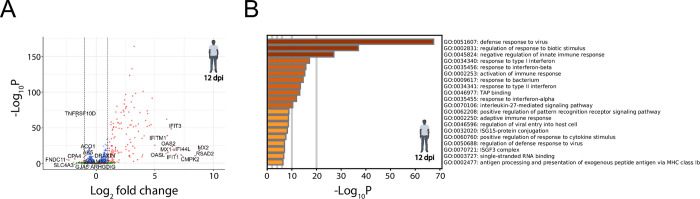
Transcriptional changes due to SEOV infection in human LMECs at 12 dpi. (**A**) Volcano plot demonstrating significantly up- and downregulated gene expressions following SEOV infection (MOI of 1.0) at 12 dpi compared to mock infection. Gene expressions are considered significantly altered when -Log_10_P ≥ 2, with 142 genes significantly upregulated (Log_2_ fold change > 1) and 10 genes significantly downregulated (Log_2_ fold change < -1). Grey dots represent genes which expressions neither significantly altered nor exceeded the fold change cut-off. Blue dots represent genes that significantly altered but did not exceed the fold change cut-off. Green dots represent genes that did not significantly alter but exceeded the fold change cut-off. Red dots represent genes that both significantly altered and exceeded the fold change cut-off. (**B**) Top 20 GO-terms identifying the most upregulated enriched pathways due to SEOV infection at 12 dpi. Biorender was used to create the image of a human.

To investigate the differences in these transcriptional gene expression profiles, differential gene expression analysis was performed. SEOV infection in human LMECs resulted in significant upregulation of 142 genes and downregulation of 10 genes at 12 dpi (Fig 3A). Upregulated genes mainly included genes involved in the interferon (IFN)-dependent anti-viral response (*MX2*, *RSAD2*, *CMPK2*, *IFIT1*, *MX1*, *IFI44L*, *OAS2*, *IFIT3*, *OASL*, *IFITM1*). Identified pathways were involved in, or related to, the defense response against viruses (Fig 3B). Significantly downregulated genes were *FNDC11*, *CPA4*, *TNFRSF10D*, *SLC4A3*, *GJA5*, *DRAXIN*, *AK5*, *ACO1*, *ARHGDIG* and *ADAM12*. Due to the small number of genes, no common enriched pathways between these genes were identified during our analyses. Taken together, SEOV-infected human LMECs displayed an interferon-focused anti-viral response at 12 dpi.

### Early anti-viral response with more immune modulation later during SEOV infection of rat LMECs

**Fig 4:**
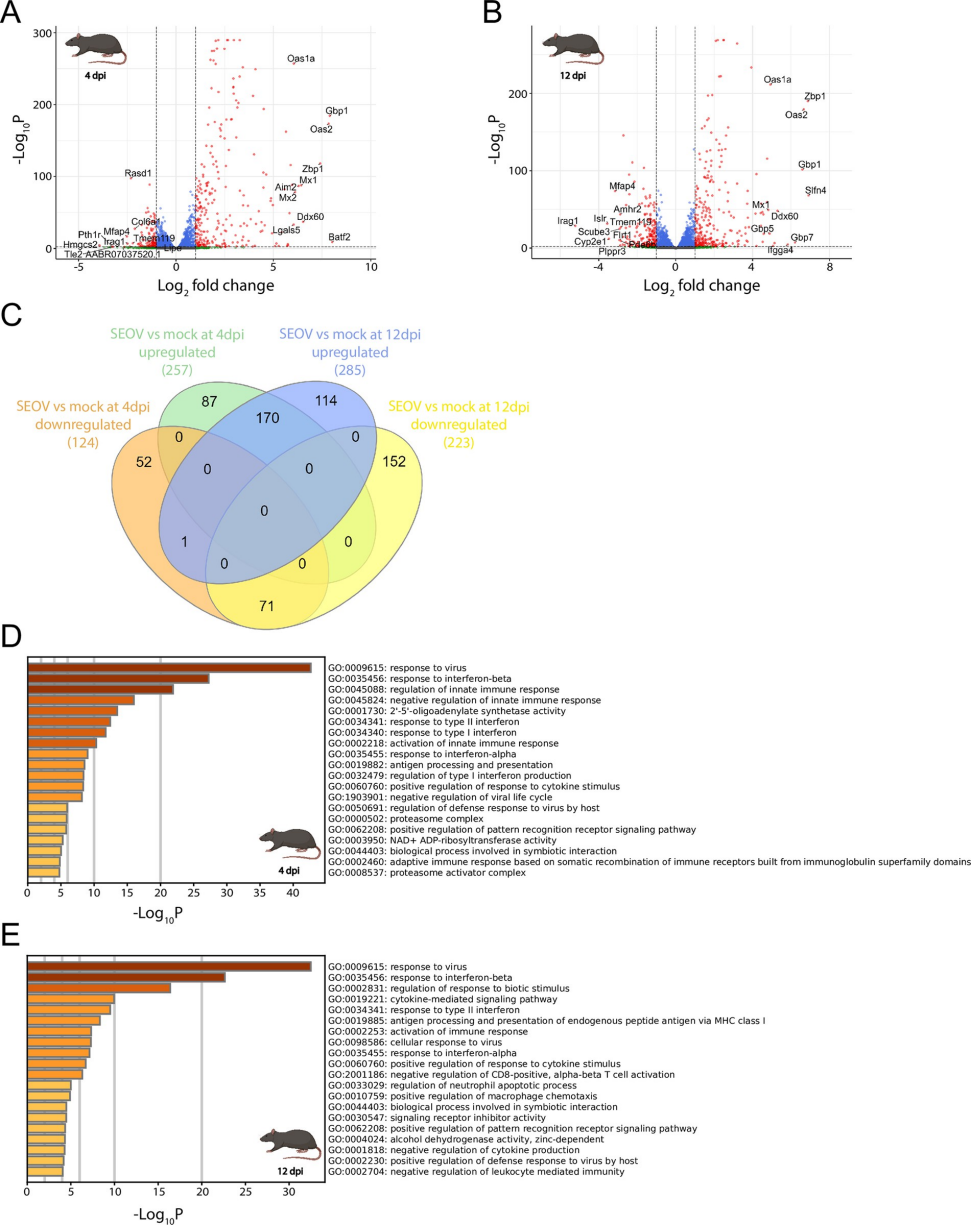
Transcriptional changes due to SEOV infection in rat LMECs at 4 and 12 dpi. (**A**) Volcano plot demonstrating significantly up- and downregulated gene expressions following SEOV infection (MOI of 1.0) at 4 dpi compared to mock infection. Gene expressions are considered significantly altered when -Log_10_P ≥ 2, with 257 genes significantly upregulated (Log_2_ fold change > 1) and 124 genes significantly downregulated (Log_2_ fold change < -1). Grey dots represent genes which expressions neither significantly altered nor exceeded the fold change cut-off. Blue dots represent genes that significantly altered but did not exceed the fold change cut-off. Green dots represent genes that did not significantly alter but exceeded the fold change cut-off. Red dots represent genes that both significantly altered and exceeded the fold change cut-off. (**B**) Volcano plot demonstrating significant up- and downregulated gene expressions following SEOV infection (MOI of 1.0) at 12 dpi compared to mock infection. Gene expressions are considered significantly altered when -Log_10_P ≥ 2, with 285 genes significantly upregulated (Log_2_ fold change > 1) and 223 genes significantly downregulated (Log_2_ fold change < -1). (**C**) Venn diagram comparing significantly upregulated and downregulated genes in rat LMECs at 4 and 12 dpi. (**D**) Top 20 GO-terms identifying the most upregulated enriched pathways due to SEOV infection at 4 dpi. (**E**) Top 20 GO-terms identifying the most upregulated enriched pathways due to SEOV infection at 12 dpi. Biorender was used to create the image of a rat.

The same analysis was performed for infected rat LMECs to identify responding genes to SEOV infection at both early and late time points. SEOV infection resulted in the upregulation of 257 genes and downregulation of 124 genes at 4 dpi (Fig 4A). Later during infection at 12 dpi, 285 genes were significantly upregulated and 223 genes were significantly downregulated (Fig 4B). Most of the significantly upregulated genes (170) were upregulated at both time points, whereas expression of 71 genes was significantly downregulated at both time points (Fig 4C). Only one gene, *Vstm2l*, was significantly downregulated at 4 dpi and upregulated later during infection at 12 dpi. At 4 dpi, the top ten significantly upregulated genes with highest fold change included *Batf2*, *Gbp1*, *Oas2*, *Zbp1*, *Ddx60*, *Mx1*, *Aim2*, *Mx2*, *Oas1a*, *Lgals5*, which are primarily involved in rat anti-viral defense responses. Similar to SEOV-infected human LMECs, identified pathways based on upregulated genes in rat LMECs were related to the defense response against viruses, response to interferon beta (IFN-β) and regulation of the innate immune response (Fig 4D). The top ten significantly downregulated genes included *Hmgcs2*, *Pth1r*, *Tle2*, *AABR07037520*.*1*, *Mfap4*, *Irag1*, *Lipe*, *Rasd1*, *Tmem119* and *Col6a1*, which are mainly involved in peptide hormone and extracellular matrix responses. Identified pathways based on downregulated genes included endothelial interactions with extracellular matrix, blood circulation, vasoconstriction and collagen binding (S2A Fig). Later during infection, at 12 dpi, the top ten significantly upregulated genes with highest fold change included, similar to 4 dpi, *Zbp1*, *Oas2*, *Gbp1*, *Ddx60*, *Oas1a* and *Mx1*. Additionally, *Slfn4*, *Gbp7*, *Ifgga4* and *Gbp5* were also highly upregulated. Although most upregulated genes were involved in pathways of anti-viral defense responses during this later time point, several enriched pathways were identified that were unique to 12 dpi and were involved in regulation of immune cells. These pathways included regulation of CD8^+^ T cell activation, neutrophil apoptotic processes and leukocyte mediated immunity, as well as macrophage chemotaxis (Fig 4E). The top ten significantly downregulated genes ranked by fold change included *Irag1*, *Islr*, *Cyp2e1*, *Mfap4*, *Flrt1*, *Scube3*, *Plppr3*, *Amhr2*, *Tmem119* and *Pde6b*. These genes were involved in extracellular matrix responses, but also in cell signaling and (lipid) metabolic processes. Enriched pathways based on downregulated genes were more diverse and included endothelial interactions with extracellular matrix, cell-cell adhesion, vasculature development, vasoconstriction and response to transforming growth factor beta (TGF-β) (S2B Fig). Collectively, these findings demonstrated a pronounced anti-viral response at both time points in rat LMECs with additional regulation of immune cells later during SEOV infection.

### Comparable anti-viral defense responses of infected human and rat LMECs

**Fig 5:**
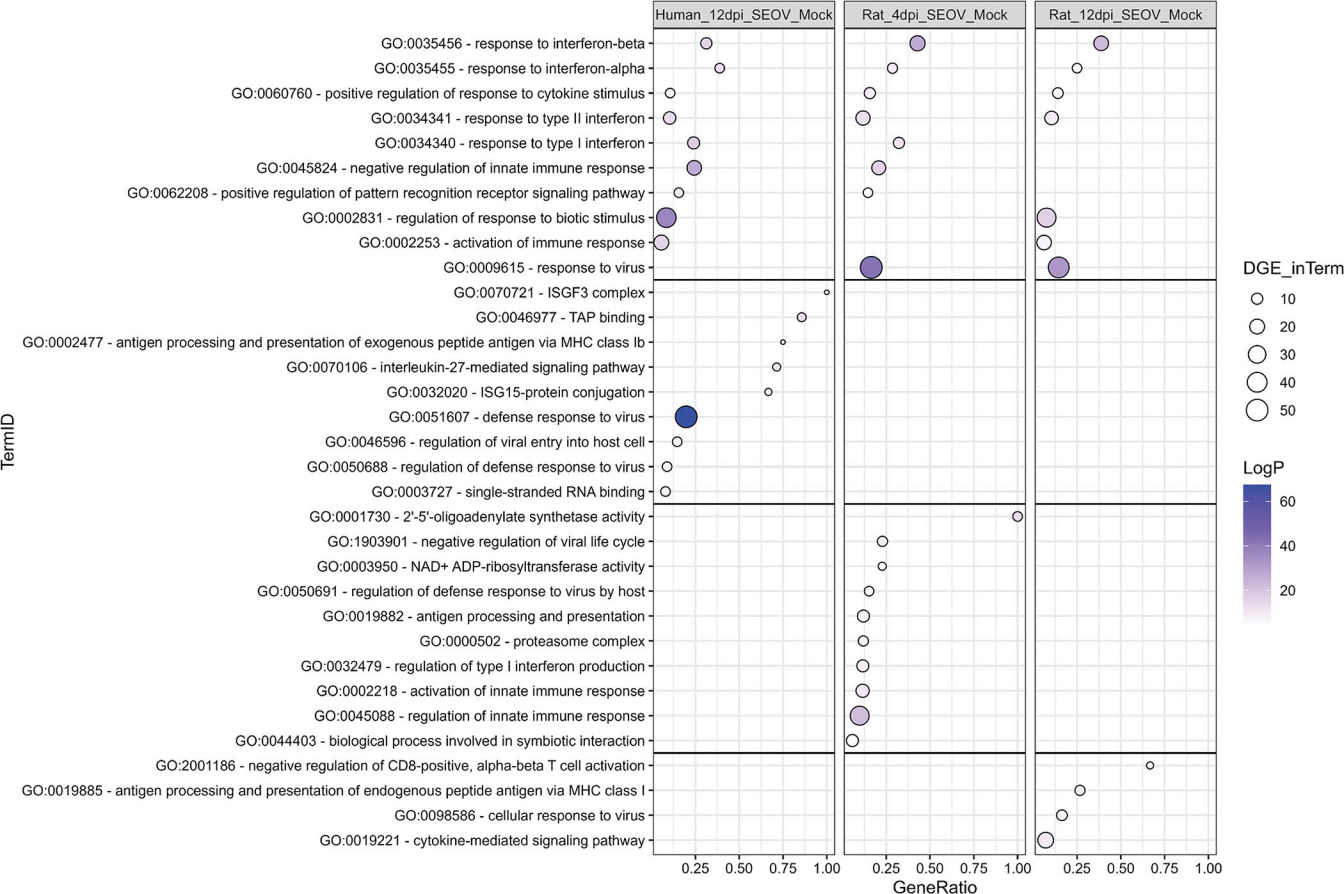
SEOV-induced transcriptomic gene expression profile overview for human LMECs at 12 dpi and rat LMECs at 4 and 12 dpi. Balloon plot displaying significantly upregulated (LogP > 5 and GeneRatio > 0.05) enriched GO-terms due to SEOV infection (MOI of 1.0) in human LMECs 12 dpi, rat LMECs 4 dpi and rat LMECs 12 dpi. Size of the balloon illustrates the number of differentially expressed genes within a GO-term (DGE_inTerm) while the color of the balloon demonstrates the statistical significance of the GO-term with a more positive LogP demonstrating a diminished chance that the observed enrichment is due to randomness. The position of the balloon on the grid displays the number of detected genes divided by the total number of genes involved in that pathway, resulting in the GeneRatio. GO-terms are ordered based on highest GeneRatio in common pathways, consequently on unique pathways for human 12 dpi, rat 4 dpi and finally rat 12 dpi.

Next, to gain more insight in differences between infected human and rat LMECs, enriched pathways during SEOV infection of LMECs were directly compared. The anti-viral state of SEOV-infected LMECs, in both species, was mainly characterized by increased expression of genes involved in positive regulation of response to cytokines, negative regulation of innate immune response, responses to type I IFN (predominantly IFN-α and -β) and type II IFN, positive regulation of pattern recognition receptor (PRR) signaling pathway and MHC-dependent antigen processing and presentation (Fig 5). These six processes cover the majority of the defense responses of SEOV-infected LMECs of both species. Other enriched pathways that were unique per experimental condition were all heavily linked to these six common anti-viral processes. However, one enriched pathway in rat LMECs at 12 dpi was uniquely linked to rather the control of excessive immune responses by negative regulation of CD8^+^ T cell activation. Taken together, these data demonstrated that the anti-viral defense response in human and rat LMECs were based on similar processes, however mediated by different genes. Although inherent species-specific differences in gene involvement between similar pathways exist, most of the genes during the anti-viral defense response were orthologous (S3 Fig).

### Specific *Cd274-*driven negative regulation of CD8^+^ T cell activation pathways in rat LMECs

**Fig 6:**
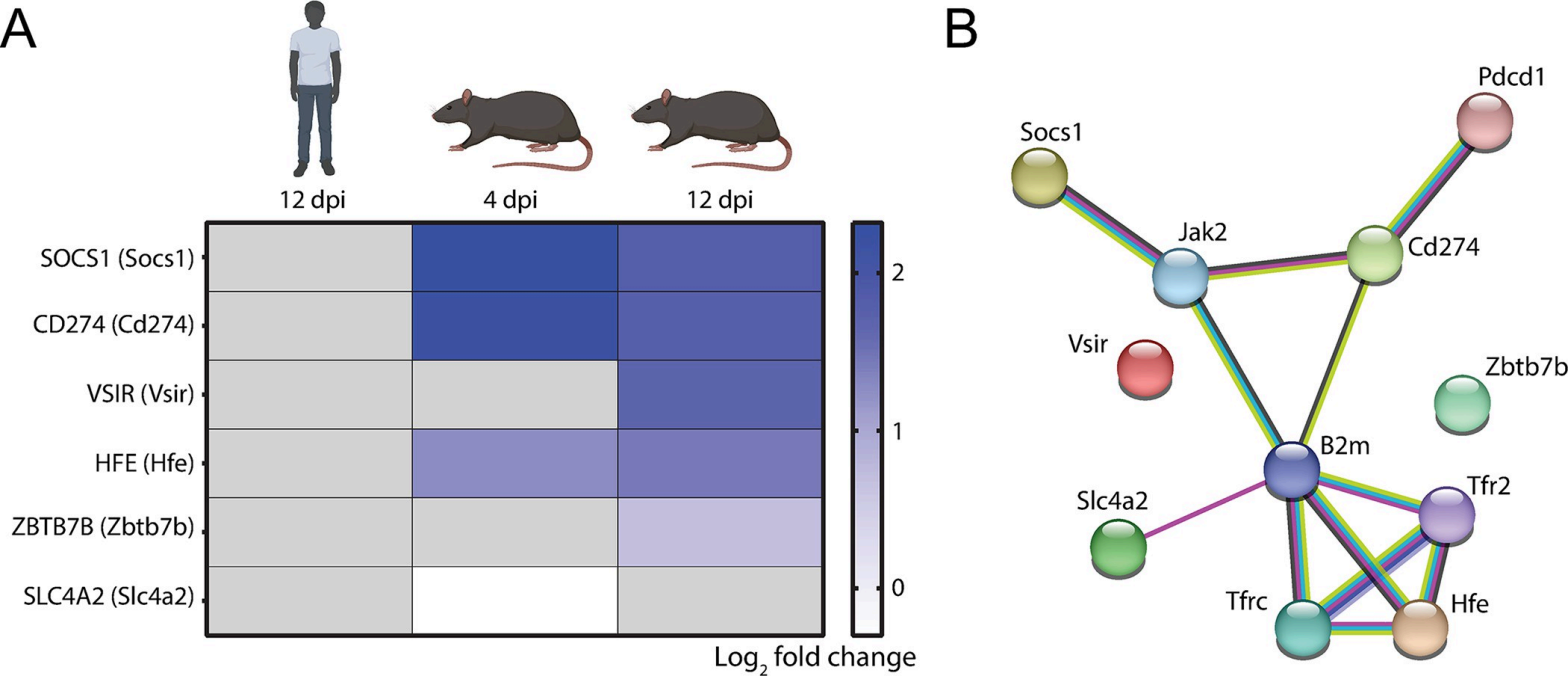
Species-specific differential gene expressions related to CD8^+^ T cell activation. (**A**) Heat map of SEOV-induced changes in expression of genes involved in negative regulation of CD8^+^ T cell activation (GO-term 2001186). Only significantly altered (-Log_10_P ≥ 2) genes are demonstrated for human 12 dpi, and 4 and 12 dpi for rat LMECs. (**B**) STRING network of protein-protein interactions involving genes playing a role in negative regulation of CD8^+^ T cell activation for rat at 12 dpi. Biorender was used for creating images of human and rat.

Since multiple studies have suggested a central role for T cells in observed immunopathogenesis in orthohantavirus-infected HFRS patients [30–34] and during emergence of persistence in reservoir rats [12,13,15], possible T cell-focused endothelial cell responses and interactions were of particular interest. Strikingly, negative regulation of CD8^+^ T cell activation was the most enriched pathway due to SEOV infection based on the Metascape GeneRatio of significantly upregulated genes specifically for rat LMECs at 12 dpi (Fig 5). Therefore, focus was placed on the underlying genes involved in this process. Although *Socs1*, *Cd274* and *Hfe* were upregulated by SEOV infection in rat LMECs at 4 dpi, *Slc4a2* was downregulated with *Vsir* and *Zbtb7b* expressions similar to mock (Fig 6A). Later at 12dpi, all genes except for *Slc4a2* were significantly upregulated in SEOV-infected rat LMECs, again suggesting that this pathway is more involved later during infection. In contrast, none of these gene expression levels were significantly altered in human LMECs at 12 dpi. However, GO-terms, as used to identify enriched pathways in this study, do not provide an endothelial cell-specific clarification for the observed negative regulation of CD8^+^ T cell activation. Therefore, these analyses were extended to a protein interaction network of which most encoding genes were expressed by rat LMECs (Fig 6B). Abovementioned genes encoded interacting proteins with a central role for programmed death-ligand 1 (PD-L1; *Cd274* expression was significantly upregulated by 2.69-fold over mock at 12 dpi), beta-2 microglobulin (B2M; *B2m* was expressed 1.93-fold over mock at 12 dpi) and Janus kinase 2 (JAK2; *Jak2* was expressed 1.32-fold decrease over mock at 12dpi).

### Confirmation of RNA-Seq results by temporal RT-qPCR

**Fig 7:**
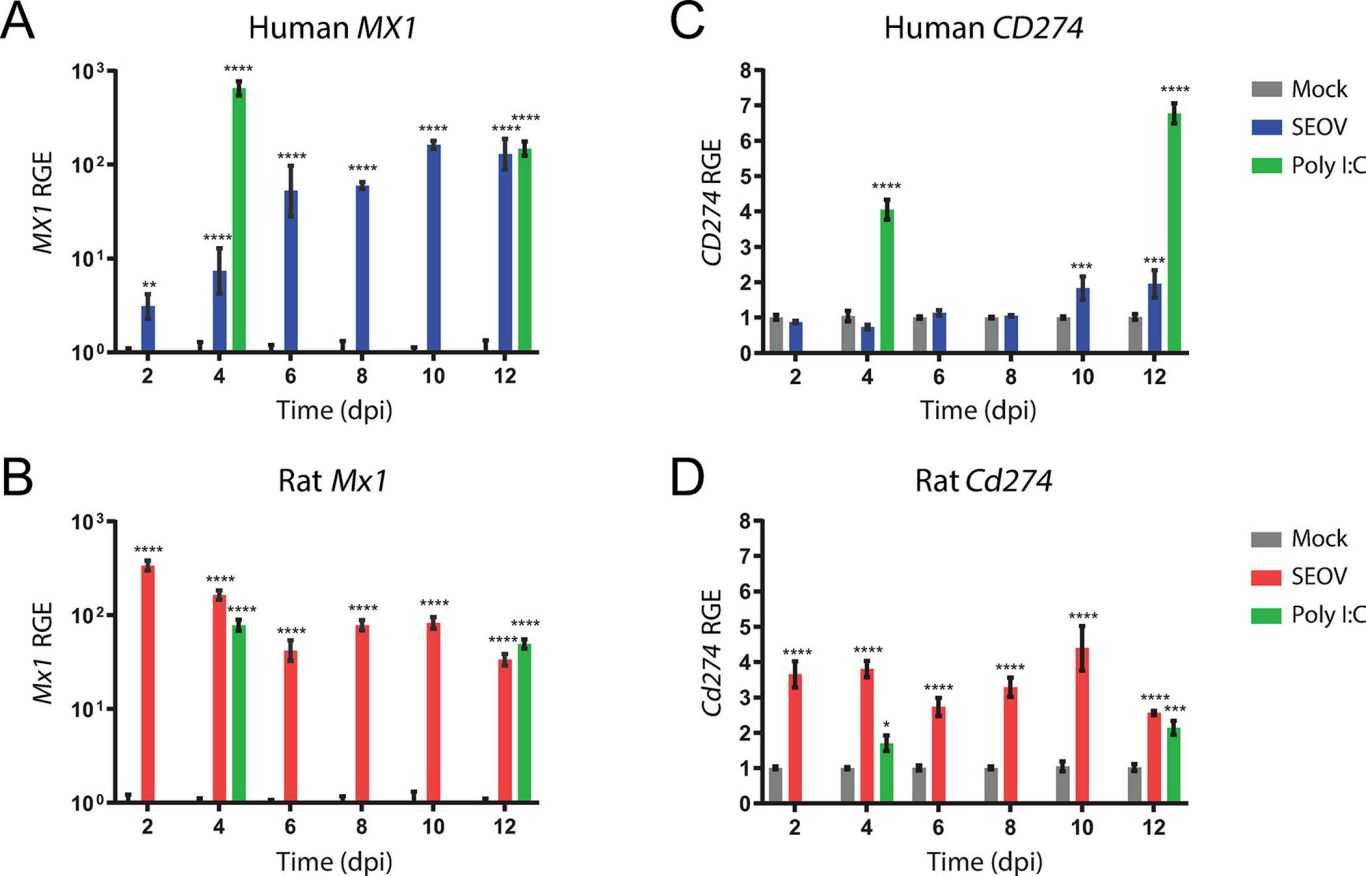
Temporal analyses and validation of RNA-Seq results via RT-qPCR. (**A**) RT-qPCR assessment of human *MX1*, (**B**) rat *Mx1*, (**C**) human *CD274* and (**D**) rat *Cd274* gene expression at 2, 4, 6, 8, 10, and 12 days after SEOV infection (MOI of 1.0). Gene expressions were normalized for species-specific housekeeping gene, human *ACTB* or rat *Actb*, and are expressed as relative gene expression (RGE) to the mock-infected condition for each corresponding time point. RGE of human *MX1* and rat *Mx1* were log-transformed. Results were compared between SEOV-infected and mock-infected cells for each time point. Poly I:C treatment (10 μg/ml for 24 hours) was included as additional control on the days of RNA-Seq and was also compared to mock-infected cells. Error bars indicate SEM with results from two independent experiments with each three independent biological replicates. Statistical significance was determined by two-way ANOVA with Dunnett’s multiple comparison test. *P < 0.05, **P < 0.01, ***P < 0.001, ****P < 0.0001.

To confirm RNA-Seq findings and get additional insight in the temporal course of gene expression levels, two prominent targets, one representing an important regulator in anti-orthohantavirus response by endothelial cells (*MX1/Mx1*, encoding MX dynamin like GTPase 1) [35,36] and a second representing the proposed immunotolerant response (*CD274/Cd274*, encoding PD-L1) [15], were investigated across different time points via RT-qPCR. The level of transcribed *MX1* gene (Fig 7A), as a surrogate for the human anti-viral response, demonstrated an increase over time corresponding with the viral replication kinetics pattern in the supernatant (Fig 1B). One of two human ortholog MX1 genes in rat, namely the *Mx1* gene expression, was taken along and peaked as early as 2 dpi. Expression levels remained high at all analyzed time points, even matching or exceeding the levels caused by the strong Toll-like receptor 3 (TLR3) activator Poly I:C (Fig 7B). Following earlier mentioned results, human *CD274* gene was not significantly increased compared to mock at each time point up to 8 dpi (Fig 7C). Significant increases were only detected later on 10 and 12 dpi, however this increase in expression levels was lower than 2-fold compared to mock, thereby remaining insufficient for reaching significance during RNA-Seq analysis. Expression of the human ortholog gene, rat *Cd274*, was significantly upregulated in rat LMECs during SEOV infection across all time points (Fig 7D). Overall, these findings confirm that the human anti-viral response was delayed, compared to the response in rat LMECs, and that the responses in rat LMECs shifted towards a less *Mx1*-directed anti-viral and a more *Cd274*-directed immunotolerant state over time.

## Discussion

SEOV infection can cause severe disease in humans while it results in a persistent infection without pathological disease in its reservoir, the rat. As in both species LMECs are an important target of infection, understanding the responses of these cells during acute SEOV infection are vital in explaining this difference in disease outcome. In this study, we characterized and compared the host responses of human and rat LMECs to SEOV infection. Current evidence suggests that in contrast to humans, SEOV infection of rats leads to virus-induced inhibition of pro-inflammatory and stimulation of regulatory responses [7,12,13,15,37]. Specifically, it has been proposed that SEOV-induced PD-L1 protein expression in rat LMECs plays a central role in this [15]. However, underlying pathways responsible for this process have neither been identified, nor directly compared between human and reservoir hosts. Hence, this study presents an extensive overview of the impact of SEOV infection on human and rat LMECs on a molecular level identifying comparable anti-viral defense responses in both species and specific regulations of T cell activation solely observed in infected rat LMECs.

Both human and rat LMECs were susceptible to infection with SEOV, which did not result in cytopathic effects. SEOV replication in human LMECs demonstrated lower infectious titers compared to rat LMECs with a lower peak of replication. This observation is in line with the relatively long incubation period of HFRS in humans which is usually multiple weeks [38]. We then moved on to perform RNA-Seq to determine the responses to SEOV in human and rat LMECs on a transcriptional level. The lack of differential genes for human LMEC samples at 4 dpi correlated with the low percentage of infected cells, which likely diluted the gene expression of infected cells so that it could not be detected by bulk RNA-Seq. SEOV infection caused an IFN-focused anti-viral response in both species with gene expression profiles in human LMECs at 12 dpi most similar to SEOV-infected rat LMECs at 4 dpi as expressions of different IFN-related rat genes were higher at this earlier time point than at 12 dpi. In line with observations for other orthohantaviruses, SEOV infection caused more upregulation than downregulation of host genes in both species [39]. Previous studies in context of HFRS have predominantly focused on anti-viral responses in human endothelial cells during Hantaan orthohantavirus (HTNV) infection. These studies also reported upregulation of multiple interferon-stimulated genes (ISGs), such as 2’,5’-oligoadenylate synthetase (OAS genes), Mx genes and IFN regulatory factors [39,40]. Comparisons between highly pathogenic HTNV, a less pathogenic species, Tula orthohantavirus, and a nonpathogenic species, Prospect Hill orthohantavirus, revealed that the ability to delay the innate IFN signaling, especially associated to induction of MxA protein, encoded by the *MX1* gene, is a crucial factor for allowing viral dissemination and thereby contributing to pathogenesis of orthohantaviruses [36,39,41]. In line with this hypothesis, in the current study *MX1* gene expression was also delayed in human LMECs upon infection with pathogenic SEOV. Alternatively, this delay might also be a consequence of delayed virus replication, rather than due to specific inhibition of *MX1* gene expression. The contrasting rapid increase in *Mx1* gene expression in rat LMECs further supports the potential role of delayed IFN signaling during the pathogenesis of HFRS. In contrast with our data, previous studies revealed lack of anti-viral responses by reservoir host cells, defined as lack of significant increase of *Mx2* gene expression, another human *MX1* ortholog, in bank vole cells as response to Puumala orthohantavirus infection [42,43]. However, these studies utilized bank vole embryonic fibroblasts, which are not target cells during natural infection, and immortalized kidney epithelial cells, which may not respond as primary cells do. Therefore, the current study, focused on a more relevant primary cell type from the specific reservoir host species, thereby possibly explaining the observed differences in anti-viral response with significant upregulations in both *Mx1* and *Mx2* expressions. Altogether, as SEOV infection and dissemination is more efficient in rat LMECs than in human LMECs, it suggests that SEOV is sensed by rat LMECs more rapidly or more efficiently, and yet the virus still manages to evade this anti-viral response as it is able to cause persistent infection in rats.

Therefore, we hypothesize that SEOV induces an early anti-viral state during acute infection in rat LMECs, but in a later phase of acute infection exploits processes for the purpose of attenuating T cell immunity. This is supported by the identification of the PD-L1-encoding *Cd274* gene, and other protein-encoding genes involved in negative regulation of CD8^+^ T cells by SEOV-infected rat LMECs. This vasculature-associated interaction network includes proteins encoded by *Socs1*, *Jak2*, *Hfe*, *B2m* and *Cd274*. *Socs1* encodes suppressor of cytokine signaling 1 (SOCS1). Endothelial SOCS1 protein leads, via JAK2 signaling, to reduction of pro-inflammatory cytokine or lipopolysaccharide (LPS)-induced endothelial cell inflammation and subsequent infiltration of immune cells, such as CD8^+^ T cells [44,45]. *Hfe* encodes a membrane protein, named hereditary hemochromatosis protein (HFE), that is structurally similar to MHC class I-type proteins and can associate with B2M, which is encoded by the *B2m* gene. Increased expression of HFE finetunes the density of MHC-I-peptide complexes at the cell surface and thereby inhibits CD8^+^ T cell activation [46]. *Cd274* encodes PD-L1 and its delicate balance with programmed cell death 1 (PD-1; encoded by *Pdcd1*) is important for restricting CD8^+^ T cell-induced immunopathology during both early and late virus infections [47]. As demonstrated during acute HTNV infection in different human cell types, PD-L1 upregulation is part of the innate immune response induced by IFNs and PRR (e.g. TLR3) signaling though not sufficient to inhibit anti-viral activity of immune cells [47–49]. A similar phenomenon may also occur during SEOV infection and could explain the observed increase in human *CD274* gene expression at later time points in this study. We hypothesize that later during acute infection in rats, the virus-induced endothelial PD-L1 increases to high levels to eliminate virus with minimal collateral vascular and tissue damage in the lungs. When virus is not entirely cleared, this results in persistent infection, as previously observed in naturally infected rats [50]. The association of severe HRFS cases with increased CD8^+^ T cell activity and initial failure to mount strong T cell responses early during acute infection, further confirms that the in rats observed PD-L1-mediated immunomodulatory mechanism is less efficient or less present in human patients, thereby failing to protect them from immunopathology [33,34]. Future studies are required to study how these proteins mechanistically work together.

A limitation to consider for this study includes that we compared host responses in cell cultures with different infection percentages. However, our data show that the difference in replication kinetics and infection percentages are due to intrinsic differences between human and rat LMECs. Therefore, utilizing the same MOI for infection and performing bulk RNA-Seq on samples at the same time points post-infection results in a more physiological representation of acute infection. This is in line with expectations that SEOV enters and replicates more efficiently in LMECs of the natural host, rats, since it has co-evolved throughout many years [51,52]. Another limitation might be the potential attenuations following propagation of SEOV on Vero E6 cells. As these changes do not influence the findings of the current study, isolation and propagation solely on physiologically relevant reservoir host or human cells should be considered for future studies. Next, due to the nature of the bulk RNA-Seq analysis, the observed lower infection percentage in human LMECs likely caused a diluting effect on the observed gene expressions. In future experiments, single cell RNA-Seq on cells with a low infection percentage should provide a more detailed overview of the differential gene expression in SEOV-infected and bystander cells. Additionally, dedicated *in vivo* infection experiments followed by spatial transcriptomics could provide further insights in cellular heterogeneity in tissues and cells responding to SEOV infection.

In conclusion, this study characterized and compared the transcriptional host responses of human and rat LMECs to SEOV infection. Both human and rat exhibit similar anti-viral responses in SEOV-infected LMECs, but with notable differences in temporal kinetics of viral replication and expression of anti-viral host genes. Rat LMECs show a unique interaction network that attenuates CD8^+^ T cell activation and potentially aids to SEOV persistence in its reservoir. These findings provide insights into host-virus interactions, HFRS pathogenesis, and pathways for viral persistence in reservoir hosts.

## Supporting information

S1 Fig Volcano plot displaying lack of significant up- and downregulated expressions of genes following SEOV infection in human LMECs 4 dpi. Upon SEOV infection (MOI of 1.0), gene expressions are considered significantly altered when -Log_10_P ≥ 2 with significant upregulation when Log_2_ fold change > 1 and significant downregulation when Log_2_ fold change < -1. Grey dots represent genes which expressions neither significantly altered nor exceeded the fold change cut-off. Green dots represent genes that did not significantly alter but exceeded the fold change cut-off. Biorender was used to create the image of a human.
(TIF)

S2 Fig Enriched pathways based on downregulated gene expressions due to SEOV infection in rat LMECs at 4 and 12 dpi. (**A**) All 18 GO-terms identifying the most downregulated enriched pathways due to SEOV infection at 4 dpi. (**B**) Top 20 GO-terms identifying the top downregulated enriched pathways based on downregulated gene expressions following SEOV infection at 12 dpi. Biorender was used to create the image of a rat.
(TIF)

S3 Fig Heat map comparing common SEOV-induced enriched pathways in human and rat LMECs based on significantly upregulated gene expressions. The heat map includes names of significantly upregulated genes responsible for the enrichment of each GO-term. Genes are considered significantly upregulated when -Log_10_P ≥ 2 and Log_2_ fold change > 1. Color of each individual cell indicates the Log_2_ fold change of each individual gene with the number of colored cells indicating the number of significantly upregulated genes responsible for each enrichment, grey cells are added for lay-out purposes. Biorender was used to create images of human and rat.
(TIF)
