# Supplementary figures and images for: Species-specific responses during Seoul orthohantavirus infection in human and rat lung microvascular endothelial cells
Source: PLoS Negl Trop Dis. 2024 Mar 27;18(3):e0012074. doi: 10.1371/journal.pntd.0012074 (PMC11020687; doi:10.1371/journal.pntd.0012074)

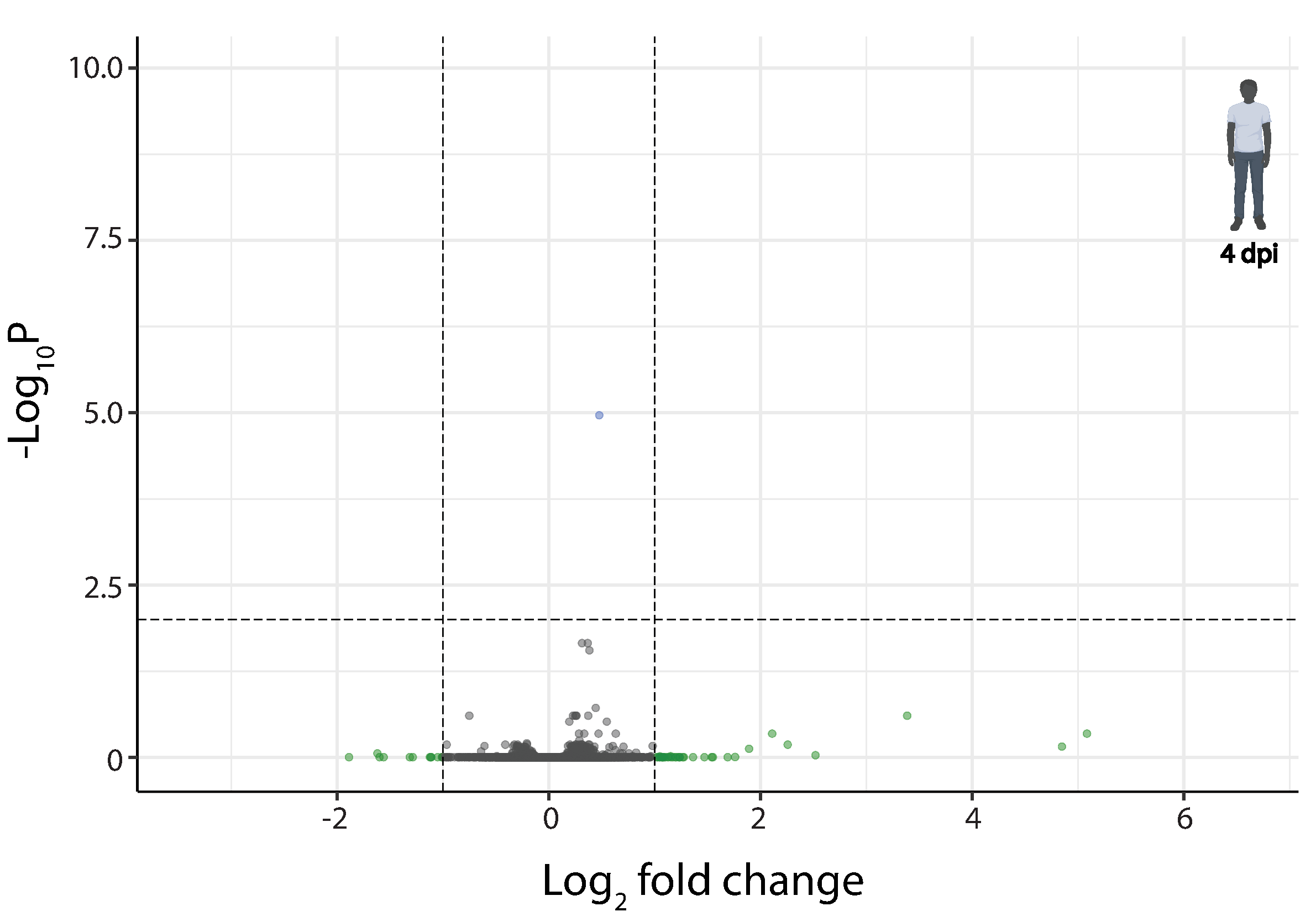

Supplement: S1 Fig — Upon SEOV infection (MOI of 1.0), gene expressions are considered significantly altered when -Log10P ≥ 2 with significant upregulation when Log2 fold change > 1 and significant downregulation when Log2 fold change < -1. Grey dots represent genes which expressions neither significantly altered nor exceeded the fold change cut-off. Green dots represent genes that did not significantly alter but exceeded the fold change cut-off. Biorender was used to create the image of a human. (TIF) [file pntd.0012074.s001.tif]

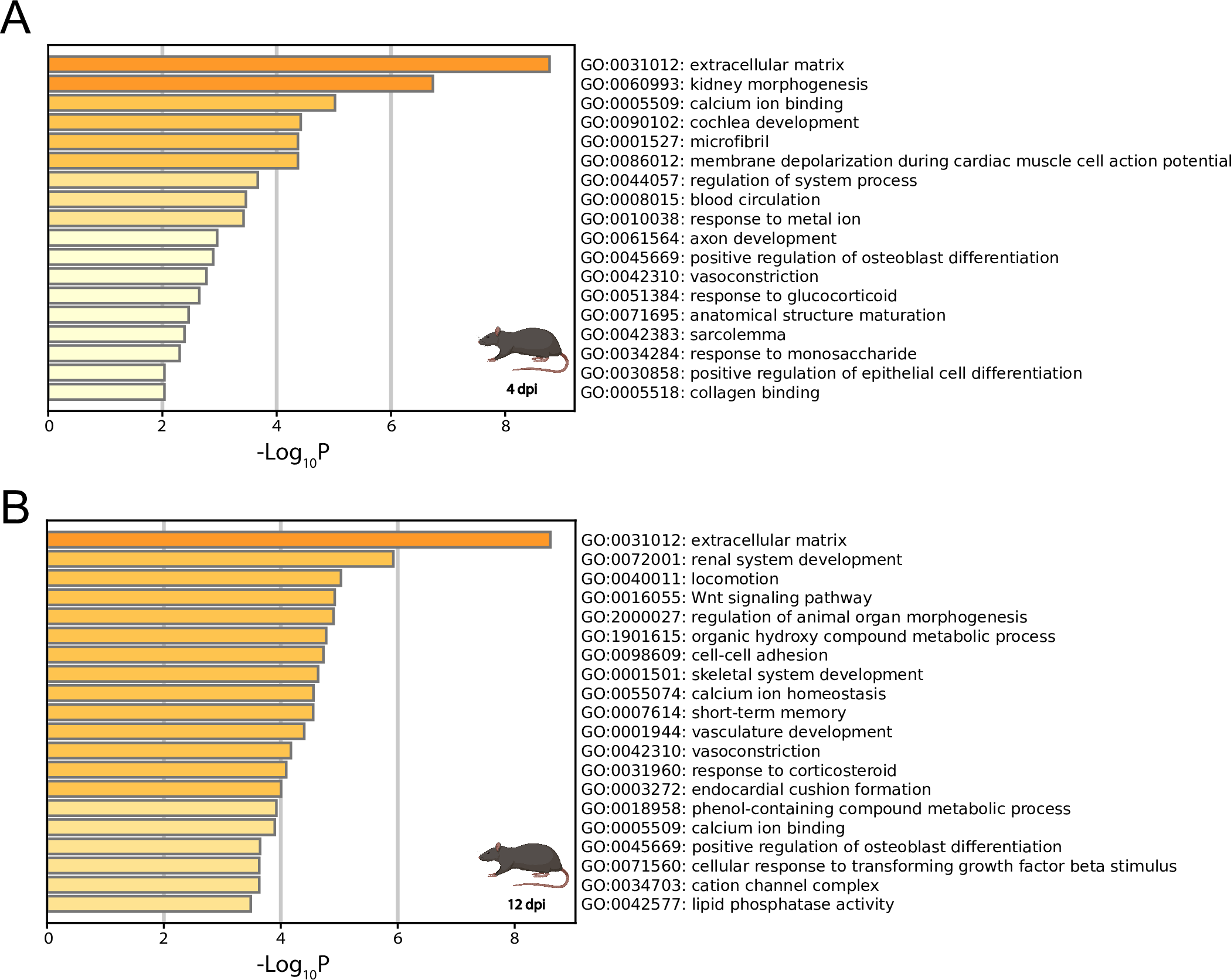

Supplement: S2 Fig — (A) All 18 GO-terms identifying the most downregulated enriched pathways due to SEOV infection at 4 dpi. (B) Top 20 GO-terms identifying the top downregulated enriched pathways based on downregulated gene expressions following SEOV infection at 12 dpi. Biorender was used to create the image of a rat. (TIF) [file pntd.0012074.s002.tif]

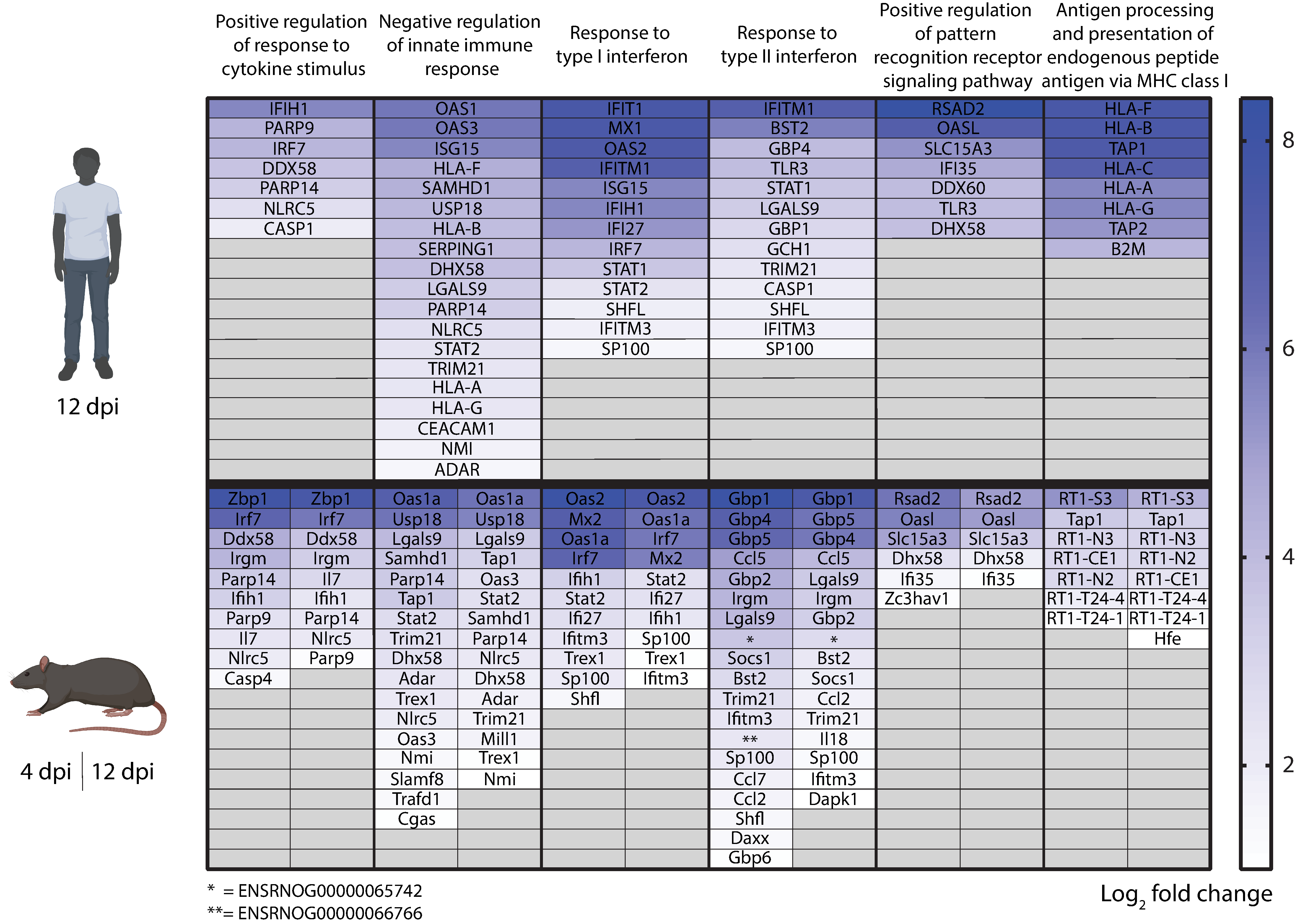

Supplement: S3 Fig — The heat map includes names of significantly upregulated genes responsible for the enrichment of each GO-term. Genes are considered significantly upregulated when -Log10P ≥ 2 and Log2 fold change > 1. Color of each individual cell indicates the Log2 fold change of each individual gene with the number of colored cells indicating the number of significantly upregulated genes responsible for each enrichment, grey cells are added for lay-out purposes. Biorender was used to create images of human and rat. (TIF) [file pntd.0012074.s003.tif]
